# Effect of Processing and Storage on the Quality of Beetroot and Apple Mixed Juice

**DOI:** 10.3390/foods10051052

**Published:** 2021-05-11

**Authors:** Flavia Bianchi, Marina Pünsch, Elena Venir

**Affiliations:** Laimburg Research Centre, Ora (BZ), 39040 Auer, Italy; flavia.bianchi@laimburg.it (F.B.); marina.puensch@gmail.com (M.P.)

**Keywords:** betalains, antioxidant activity, colour, pasteurized juice, thermal treatment, shelf-life

## Abstract

In recent years, there has been a growing interest in the development of health-promoting and disease-preventing functional foods. Beetroot is a promising vegetable because of its outstanding antioxidant activity, vivid colour, and content of bioactive compounds. In the present study, the quality of pure beetroot and apple juices as well as that of their mixture was evaluated by measuring changes of colour, betalain content, and antioxidant activity during processing and storage. No perceivable colour changes of the beetroot juice were observed after adding apple juice up to 85% of the total amount. However, the antioxidant activity was proportionally reduced with the addition of apple juice. Pasteurization negatively affected the content of betalains but did not influence the antioxidant activity. Storage led to colour modifications and reduction of betalains and antioxidant activity. Through preliminary shelf-life studies, a durability of 65 days at room temperature for apple and beetroot juice blends was calculated. The novelty of this study lies in an extended description of physico-chemical characteristics of a fresh apple and beetroot blended juice obtained from local products, in the study of the effects of processing and storage on its quality, and in the estimation of its shelf-life after storage at different temperatures.

## 1. Introduction

Beetroots (*Beta vulgaris*) are a good source of dietary fibres, minerals, vitamins, phenolics, and antioxidant compounds [1,2,3,4,5,6]. They have a potential relevance for human health, since they provide a high content of functional components known for their antioxidant and anti-inflammatory properties [7,8] and anticarcinogenic potential [9,10,11].

A unique characteristic of beetroot is its intense colour, due to the presence of betalains, hydrosoluble pigments, that gained increasing attention because of their antioxidant, anti-inflammatory, and chemo-preventive effects [8,12,13,14,15,16,17,18]. The mechanism at the basis of the antioxidant capacity of betalains is related to the activity of their core of betalamic acid: through the direct reduction of two Fe^3+^ ions to Fe^2+^, this compound is able to donate two electrons to an oxidizing agent [18]. Additionally, the chemical structure of betalains influence their antioxidant potential: the presence of an aromatic ring positively affects the radical scavenging activity; in betaxanthins, the antioxidant capacity depends on the number of hydroxy and imino residues, while acylation and glycosylation lead to an increased or decreased antioxidant activity of betacyanins, respectively [17].

Betanin, the most abundant betalain in beetroot, was reported to inhibit lipid peroxidation and tumour cell proliferation [19].

Besides betalains, other beetroot compounds are reported as bioactive, including nitrate, betaine, ascorbic acid, carotenoids, polyphenols, flavonoids, glycine, and folate [14] and rutin, epicatechin, and caffeic acid, which also exhibit antioxidant activity [20].

Numerous studies reported the positive effect of beetroot juice supplementation on the protection against lipid and protein oxidation [8,15,16]. The World Health Organization, for “diet, nutrition and the prevention of chronic diseases”, suggests a minimum daily intake of fruit and vegetables for adult people of 400 g [21]. Nevertheless, data from Eurostat show that only 14.1% of the European population reach the recommended daily intake of five or more portions of fruit and vegetables [22]. A possibility to enhance the intake of fruits and vegetables in the diet is the assumption of functional beverages. Recent research demonstrated the higher amount of phenolics and flavonoids as well as a better antioxidant profile of beetroot juice (based on the stable free radical, 2,2-diphenyl-1-picrylhydrazyl (DPPH) assay) compared to citrus fruits, yellow passion fruit, apple, and cranberry [23].

Although beetroot juice provides many health benefits, from the point of view of consumers, the presence of geosmin, which impart the typical earthy flavour to beetroots, negatively affects the sensory characteristics of food products with added beetroot juice [24]. According to previous studies, beetroot juice makers are encouraged to improve the taste and odour of their products, in order to satisfy consumers’ liking [25]. Flavour, colour, nutritional, and organoleptic characteristics of a fruit juice are very important for consumers and were often found to be superior in blended juices compared to juice prepared from individual fruit, indicating that a blended beverage containing fruit and vegetables can better satisfy consumers’ preferences [26]. Positive sensory evaluations were reported concerning formulations prepared adding fruits and/or spices to beetroot juice, including orange, ginger, and carrot [27], or apple and carrot [28], or ginger and ehuru [29], highlighting how blending can improve sensory quality of beetroot juice. Based on this background knowledge, it would be recommended to consider mixing beetroot juice with other fruits or vegetables in order to meet consumers’ taste.

Besides the importance to achieve a good acceptance by consumers, it is equally relevant that the functional compounds present in such beverages are preserved during processing and storage [13]. Parameters such as pH, temperature, oxygen, and light exposure influence the pigments stability and thus the colour of the final product.

Due to the high pH of beetroots, strategies to ensure microbiological stability, such as storage at low temperatures for short time, or addition of acidic ingredients, are necessary [30,31,32]. Though the high pH represents a disadvantage concerning the microbiological stability of beetroot juice, the presence of betalains provides an advantage concerning the stability of blended juices, obtained through mixing of beetroot juice with more acidic fruits or vegetables. Indeed, betalains are reported to be more stable to temperature and to a wider pH range than commonly used natural food colourants, like anthocyanins [31].

In the present study traditional, local South Tyrolean agricultural goods were utilized to produce a beverage based on beetroot and apple juice. The beverage was designed to respond to stability criteria, thanks to the replacement of common acidifiers with apple juice [33]. The choice of apple juice was not only related to the wide production of apples in South Tyrol, but also to the fact that these fruits represent themselves a good source of bioactive compounds, including flavonoids and phenolics, and possess high antioxidant activity [34,35].

To our knowledge, only a study from 2020 investigated the effects of storage on pH, soluble solids, colour, betalains, and 5-hydroxymethylfurfural in a blended apple and beetroot juice [36], pointing out that only limited information concerning such a fruit and vegetables blended juice is available, and indicating, at the same time, a recent interest of research on its quality and shelf-life.

The originality of the present work relies on the durability study and on the evaluation of processing on the quality of a beetroot and apple mixed juice, whose literature data are scarce.

Thus, the aim of this research was to investigate the effect of the processing steps required for the preparation of an apple and beetroot blended juice (extraction, thermal treatment, and blending) on pH, soluble solids, colour, betalains, and antioxidant activity and to estimate a provisional shelf-life of such juice.

## 2. Materials and Methods

### 2.1. Apple Juice and Pasteurized Apple Juice

A fresh commercial cloudy apple juice (AJ) was purchased from a local producer in Laces, South Tyrol. The AJ consisted of a pasteurized juice containing 100% apples. The juice was bottled in 90 mL glass jars. Four jars were stored at −30 °C until analyses, while other 66 jars were subjected to further pasteurization (PAJ) at 85 °C for 3 min after the temperature in the core reached 85 °C, in an oven with steam function (CM 61, Rational Italia S.r.l., Mestre, Italy). Subsequently, the product was cooled to room temperature with ventilated air. The process did not exceed 8 min. Pasteurized samples were stored at ambient temperature in dark conditions for defined ranges of time and then kept at −30 °C until analysis.

### 2.2. Beetroots and Beetroot Juice

‘Detroit Dark Red’ variety beetroots were purchased from a local producer in Laces, South Tyrol, in October 2019. Beetroots were sorted and washed thoroughly with cold water, removing all soil residues.

An aliquot of raw vegetables was immediately stored at −30 °C, defrosted in February 2020, and the juice was extracted using a household juice extractor. The fresh beetroot juice obtained (FBJ) was stored at 4 °C for 3 h for decantation, the upper phase was collected, and the juice was stored at −30 °C until further processing and analysis.

An aliquot of beetroots was immediately processed into juice using an industrial pilot plant. The juice extraction was carried out using a turboextractor (Multipass 250, Vemia S.r.l., Parma, Italia), equipped with 0.5 mm cut-off mesh. The temperature of the outlet product was approximately 47 ± 2 °C. The cloudy juice was stored at 4 °C for 3 h for decantation, and the upper phase was collected. The beetroot juice (BJ) was stored at −30 °C until further processing and analysis.

Part of the BJ was further subjected to pasteurization using the same conditions reported for PAJ, obtaining a pasteurized beetroot juice (PBJ), stored at −30 °C.

Prior analysis all beetroot-containing juices were subjected to centrifugation (Sorvall ST 16 R Centrifuge, Thermo Fisher Scientific, Waltham, MA, USA) at 1500 g at 15 °C for 10 min to remove suspended particles.

### 2.3. Beetroot and Apple Mixed Juice

The optimal percentages of apple juice (85%) and beetroot juice (15%) were based on previous results [33], to ensure that the final pH of the mixed juice was lower than 4.6, as recommended for microbiological safety reasons [37]. For the beetroot and apple mixed juice (MIX), 5 L of AJ were mixed with 0.9 L of BJ and left overnight at 2 °C for decantation. The upper phase was collected and bottled in 90 mL glass jars. Four jars were stored at −30 °C until analysis. The remaining 66 jars were pasteurized (PMIX) using the same conditions reported for PAJ. The process did not exceed 8 min. Pasteurized samples were stored at ambient temperature in dark conditions for defined ranges of time and then kept at −30 °C until analysis.

### 2.4. Sugar Content

The sugar content was reported as °Brix and measured using a portable LCD digital refractometer (PAL-α, Atago, Tokyo, Japan). Measurements were performed in triplicate on AJ, PAJ, FBJ, BJ, PBJ, MIX, PMIX at 20.0 ± 2 °C, and the refractometer prism was calibrated with distilled water prior to analysis.

### 2.5. Dry Matter

Dry matter (DM) was determined gravimetrically, following the AOAC vacuum drying method 44.1.03A [38]. Water was evaporated at low temperature (65 °C) in a vacuum drying chamber (VD 53, BINDER Inc., Bohemia, NY, USA) under vacuum conditions (2 mbar) for at least 12 h. The tare of the weighing boats was determined at 25.0 ± 2 °C using an analytical balance at four decimal points (Explorer EX255D, OHAUS Europe GmbH, Nänikon, Switzerland) after approximately 4 h drying at 65 °C under vacuum conditions to remove residual moist. Briefly, 2 mL of sample was transferred in the weighing boat and the exact weight was recorded. The samples were dried in the drying chamber at 65 °C at atmospheric pressure until samples were viscous enough (approx. 5 h), before setting the vacuum to 2 mbar overnight. The dried and warm samples were cooled at room temperature in a glass desiccator containing silica gel, and then, the weight was recorded. Measurements were performed in triplicate on AJ, PAJ, FBJ, BJ, PBJ, MIX, PMIX.

### 2.6. pH

The pH of all samples was determined at 20.0 ± 2 °C using a digital pH meter (Orion Star A221, Thermo Fisher Scientific, Waltham, MA, USA). The device was calibrated using standardized pH buffers of pH 4.00 and 7.00 prior to analysis. Measurements were performed in triplicate on AJ, PAJ, FBJ, BJ, PBJ, MIX, PMIX.

### 2.7. Colour Measurement

The colour of each sample was determined at 20.0 ± 2 °C after transferring part of the sample in a glass Beaker, by measuring the CIE colour values (L*, a*, and b*) using a colourimeter (CR-400, Konica Minolta, Tokyo, Japan). The colourimeter was calibrated before each analysis. The total colour difference, expressed as ∆E, was calculated. Six measurements were taken on each sample of beetroot-containing juices (FBJ, BJ, PBJ, MIX, and PMIX).

### 2.8. Betalains

The quantification of betaxanthins and betacyanins in the beetroot-containing juices (BJ, FBJ, PBJ, MIX, and PMIX) was determined spectrophotometrically in a citrate-phosphate buffer, according to the method reported by Stintzing et al. [6] and further modified by Sawicki et al. [39], using a UV-VIS spectrometer (UV-1800, Shimadzu, Kyoto, Japan). Samples were diluted in citrate/phosphate buffers at pH 4.5 and 6.5 [40] until absorption values in the optical density (OD) ranged between 0.8 and 1.2 at 538 nm and at 480 nm for betaxanthins and betacyanins, respectively. Disposable cuvettes (1 cm optical path length) were used, and the analysis was performed at 25 °C. The betalain content (BC) was calculated according to Lambert–Beer’s law. The betacyanin concentration was calculated using the molecular weight (550 g/mol) and molar extinction coefficient (60,000 Lmol^−1^cm^−1^ in H_2_O) of betanin, considered as representative for betacyanins [6]. The betaxanthin concentration was calculated using the molecular weight (308 g/mol) and molar extinction coefficient (48,000 Lmol^−1^cm^−1^ in H_2_O) of indicaxanthin I, considered as representative for betaxanthins [6]. Six measurements were taken on each sample.

### 2.9. Antioxidant Activity

The antioxidant activity (AA) of the juices was determined spectrophotometrically according to the method of Brand-Williams, Cuvelier, and Berset [41], modified by Manzocco et al. [42], as radical scavenging activity using the DPPH assay. The DPPH solution was prepared by dissolving DPPH powder in methanol until an absorbance value at 515 nm (OD_515_) was in the range between 1.0 and 1.6. A volume of 2.9 mL of DPPH solution and 10 µL of juice for all samples containing only beetroot (FBJ, BJ, and PBJ) were used. For all the other juice types (AJ, PAJ, MIX, and PMIX) the volume used was 20 µL in 2.9 mL DPPH solution. The kinetic of bleaching, in which the absorbance of the solution was recorded at 515 nm at 25 °C for 20 min with one measurement per minute, was recorded. The antiradical activity (AA) was normalized based on the dry matter in the cuvette and expressed as bleaching rate per mg of dry matter [-OD_515_min^−1^mg_DM_^−1^]. For beetroot-containing juices, the AA was also normalized based on the total betalains. Six measurements were taken on each sample.

### 2.10. Accelerated Shelf-Life Test

For accelerated shelf-life testing, the PMIX was incubated at 30, 40, and 50 °C for 25 days. Sampling was performed at 0, 15, and 25 days for storage at 30 °C and 40 °C and 0, 7, 15, and 25 days for storage at 50 °C.

### 2.11. Data Elaboration and Statistics

Independent *t*-tests were performed, with a significance level of *p* = 0.05, to evaluate differences between samples. Pearson correlation coefficients (r) and linear regression lines were calculated to evaluate correlations between colour, total betalains, and antioxidant activity. The Arrhenius plot was used to predict the reaction rate at lower temperatures compared to the investigated ones [43]. Statistical elaboration and data visualization were performed using SPSS (IBM SPSS Statistics 24) and Excel (Microsoft Office 16).

## 3. Results

### 3.1. Effect of Processing

A summary of the dataset is reported in Table 1.

The impact of different processing steps on basic analytical parameters as well as on defined quality parameters (colour, betalain content, and antioxidant activity) of the different juices was investigated. Samples were compared in pairs to assess the effect of each processing step, as shown in Table 2.

The results concerning the basic analytical parameters (pH and sugar content) are summarized in Table 3.

The dilution of BJ with AJ to a concentration of 15% resulted in a successful reduction of the pH below 4.5, ensuring microbiological safety. PBJ exhibited a lower pH with respect to BJ. The decrease in pH after thermal treatment of beetroot juice has already been reported and addressed to the degradation of ascorbic acid [32].

The sugar content of BJ was found to be lower than the one of AJ. Consequently, the sugar content of the MIX was lower to that of AJ. The highest sugar content was observed in the juice extracted from the beetroots which were harvested and immediately frozen at −30 °C (FBJ), whereas the sugar content of BJ, which was extracted from fresh raw vegetables and then stored for 4 months at −30 °C, was significantly (*p* < 0.05) lower. The difference between FBJ and BJ is not ascribable to raw material, since the beetroots belonged to the same batch. It concerned, instead, the extraction step. It would be expected that the rise of the temperature up to 47 ± 2 °C during the extraction of BJ would lead to a higher sugar extraction compared to FBJ. Differently from expectations, FBJ contained more sugars, indicating that freezing prior extraction affected the sugar yield in the juice. Indeed, sugars in beetroot tissue are mainly compartmentalized in cell vacuoles, free space, and cytoplasm [44]. Slow freezing leads to the formation of large ice crystals in the extracellular area, which can damage the cell wall and break the cell membrane [45]. The freezing of the fresh beetroots could have caused a higher degree of tissue damage, with a consequently higher extraction of sugars located into those organelles.

#### 3.1.1. Colour

Appearance, and thus colour, is among the major attributes concerning the quality of fresh and processed food products, and influences consumer’s choices, preferences, willingness to purchase, and perception of healthfulness [46,47,48]. Concerning beetroot juice, this parameter is affected by the content of pigments, especially betalains, as well as processing steps, including thermal treatments [49].

The effects of processing on juice colour were investigated using CIE colour parameters (L*, a*, and b*). The slightly yellowish or brownish colour of AJ was covered by the red/violet colour of BJ; hence, only the colour of the juices containing beetroot were analysed. The lightness (L*), redness (a*), and yellowness (b*) of the samples at different processing stages are reported in Table 4.

No significant difference in colour between BJ and MIX was observed, despite the lowering of pH after the addition of AJ.

The colour of BJ and MIX was affected by thermal processing. A significant (*p* < 0.05) increase of L* was observed comparing PMIX and MIX. There was a significant increase (*p* < 0.05) in a* values after pasteurization (∆a* of 5.15 ± 1.22) between MIX and PMIX. It is known that betacyanins, the red-violet pigments of beetroots, are more stable than yellow betaxanthins [12,30,50]. The change of b* value between MIX and PMIX (∆b* of 1.24 ± 0.37) was found to be not as high as that of a* value but still significant (*p* < 0.05).

The total colour difference, expressed as ∆E, was calculated to assess the change in visual perception of the juice colour during processing. Results are shown in Table 5.

Extraction (FBJ-BJ) through the use of a turboextractor caused only little colour variation even though the juice reached a temperature of approximately 47 °C, indicating a relatively stable juice to mild temperature exposition for short-medium time.

The blending step (BJ-MIX) led to the smallest change in total colour difference, considered as not perceptible by human eyes. This was surprising, since the dilution of BJ with a high volume of AJ reduced the pH, an influencing factor for betalain structure and thus, colour stability.

The pasteurization step affected the juice colour. A ∆E of 5.38 observed between MIX and PMIX is considered as perceptible at a glance.

#### 3.1.2. Betalains

Besides their potential beneficial effect on human health [7], betalains are the pigments responsible for the vivid colour of beetroot [51]. Given the importance of colour for beetroot juice, it is crucial to study the effect of juice processing and storage on betalain content.

**Figure 1:**
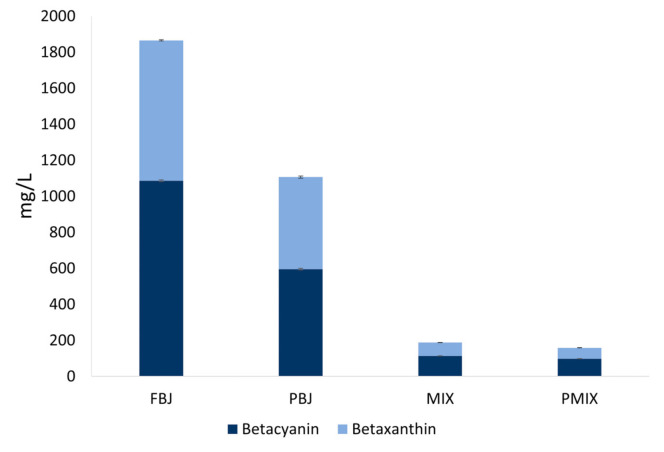
Betalain content (betaxanthins and betacyanins) in fresh beetroot juice (FBJ), pasteurized beetroot juice (PBJ), beetroot and apple mixed juice (MIX), and pasteurized beetroot and apple mixed juice (PMIX).

The impact of processing of BJ on the betalain composition and the total betalain content was investigated. Betaxanthin and betacyanin contents during the different processing steps are reported in Figure 1.

Extraction (FBJ-BJ) affected the betalain content: the highest betalain content was observed in FBJ (1865.18 ± 5.45 mg/L), whereas in BJ betalain content was much lower (1057.69 ± 61.38 mg/L). This difference can be explained by the fact that betalain pigments are mainly stored in the cell vacuoles of the plants that synthesize them [52]. As already observed for sugar content and dry matter, the freezing process of the fresh beetroots could have led to higher degree of tissue damage and thus to an increased extraction of betalains, as reported by Verberic et al. [53] for anthocyanins.

The blending step (BJ-MIX) of beetroot juice with apple juice, which does not contain betalains, caused a reduction of total betalains by 82%, which is proportional to the dilution ratio. In fact, the betacyanin and betaxanthin content of MIX (114.21 ± 0.75 mg/L and 73.52 ± 0.63 mg/L, respectively) were lower than those of the juices containing only beetroot.

The pasteurization step on MIX led to a significant (*p* < 0.05) reduction of betalains: both betacyanins and betaxanthins were lower in the pasteurized juice (98.13 mg/L versus 114.21 mg/L for betacyanins and 60.41 mg/L versus 73.52 mg/L for betaxanthins), leading to a reduction of 16% in total betalain content (187.73 mg/L before, versus 158.54 mg/L after pasteurization). As expected, based on literature data [13], the reduction of betaxanthins was found to be higher (18%) than the reduction of betacyanins (14%) after thermal treatment.

#### 3.1.3. Antioxidant Activity

The high antioxidant activity of beetroot, which indicates their potential use in human diseases [7], is of great importance for the quality of a functional beverage based on these vegetables.

**Figure 2:**
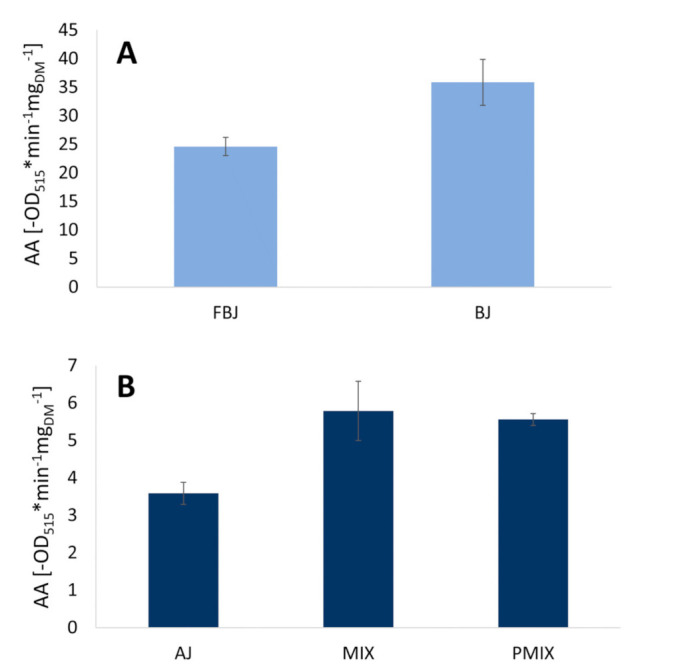
Antioxidant activity (AA) of juice samples. (**A**) Comparison of the AA of fresh beetroot juice (FBJ) and beetroot juice (BJ), showing the effect of the extraction. (**B**) Comparison of the AA of apple juice (AJ), beetroot and apple mixed juice (MIX), and pasteurized beetroot and apple mixed juice (PMIX), showing the effect of the blending and of the pasteurization.

The results concerning antioxidant activity of juice samples after processing steps are shown in Figure 2.

The extraction (BJ-FBJ) through the use of a turboextractor lead to a significant (*p* > 0.05) increase of the antioxidant activity in BJ with respect to FBJ. Calculations were carried out, assuming that the whole dry matter of the samples (DM% of FBJ = 15.392 ± 0.002; DM% of BJ = 10.096 ± 0.003) had antiradical activity; thus, the higher dry matter of FBJ could have led to an artifact. The antioxidant activity was recalculated based on the betalain content instead of the dry matter. Based on this calculation, BJ was characterized by an even higher antioxidant activity with respect to FBJ when calculated per mg of betalains, with values of 3422.57 ± 382.42 -OD_515_*min^−1^mg_BC_^−1^ and 2030.19 ± 131.58 -OD_515_*min^−1^mg_BC_^−1^ for BJ and FBJ, respectively. This indicates a positive effect of turboextraction on the availability of antiradical potential of betalains, probably due to the temperature rise. As reported by Ravichandran et al. [31], thermal treatment of red beet products leads to a decrease of betalains coupled with an increase in antioxidant activity, which could be explained by the presence of other compounds with antioxidant potential like polyphenols and vitamins, which act synergistically but are differently sensitive to temperature and processing. Furthermore, it is known that polymerized forms of phenolic compounds show better antioxidant properties than their monomers [41]; thus, it can be inferred that warming during turboextraction could have led to partial polymerization of monomers, which in turn resulted in higher antioxidant activity.

Since the antioxidant activity of BJ was found to be 10 times higher than the potential of AJ (35.84 ± 4 -OD_515_*min^−1^mg_DM_^−1^ versus 3.59 ± 0.3 -OD_515_*min^−1^mg_DM_^−1^, respectively), the blending step resulted in an increase of the AA of MIX (7.79 ± 0.79 -OD_515_*min^−1^mg_DM_^−1^) compared to AJ and a decrease compared to BJ.

Pasteurization (MIX-PMIX) did not significantly affect (*p* > 0.05) the antioxidant activity.

### 3.2. Preliminary Accelerated Shelf-Life Test

Results concerning the shelf-life of PMIX should be considered as a preliminary study, since the processing of the product analysed was only carried out over a short period and in a pilot plant but not on an industrial scale, as recommended [54].

The effect of storage at different temperatures (30, 40, and 50 °C) for 25 days on colour, betalain content, and antioxidant activity of PMIX was investigated.

**Figure 3:**
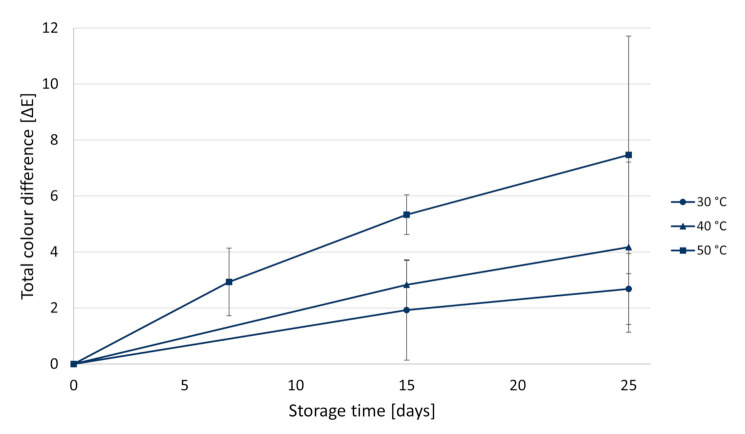
Total colour difference (∆E) between the colour of pasteurized apple juice (PMIX) immediately after processing and that of juices stored at different temperatures and collected at different time points over a storage period of 25 days.

Concerning the quality parameters investigated, colour was the one mostly affected by storage. Since this parameter is highly relevant for customers purchasing decision [46], shelf-life estimation was based on colour changes. Results concerning the colour change (expressed as ∆E) of PMIX stored at different temperatures (30, 40, and 50 °C) over 25 days are shown in Figure 3. As expected, a higher colour change was observed at higher temperatures.

**Figure 4:**
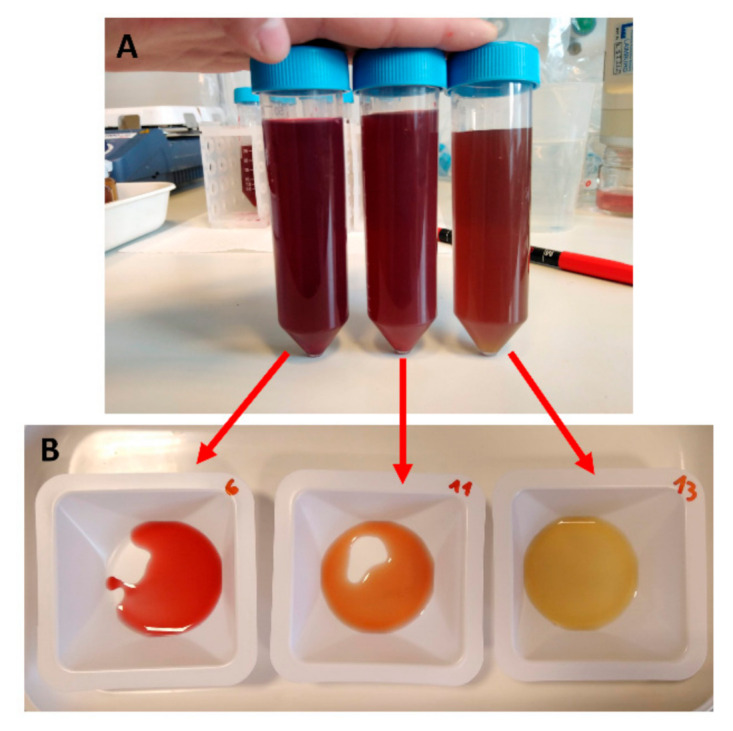
Pasteurized beetroot and apple mixed juice (PMIX) after 15 days of storage at 30, 40, and 50 °C (from left to right), (**A**) in centrifuge tubes, (**B**) and in weighing boats.

Through visual inspection, after 7 days of storage, all samples exhibited similar colour. PMIX stored at 30° did not show visible colour change during the whole experimental period (∆E 2.68 ± 1.26). On the contrary, after 15 days, the colour of the sample stored at 50 °C was visibly different (Figure 4), as confirmed by a ∆E value of 5.33 ± 0.71. For the juice stored at 40 °C, visible changes of colour (∆E 4.17 ± 3.03) could be observed after 25 days. Colour changes were more evident when small amounts of samples were put in a white weighing boat (Figure 4B). However, the difference was not so clear when centrifuge tubes were visually examined (Figure 4A).

Based on these observations, it was assumed that a ∆E > 4 was clearly visible and thus considered as the “technological” threshold value for the shelf-life of the product. Nevertheless, the slightly bleached juices could still be acceptable. Thus, a sensory or a consumer test could be further performed, in order to ascertain whether this colour change would be still acceptable by consumers. In this case, the “sensory” shelf-life could be longer than that reported.

**Figure 5:**
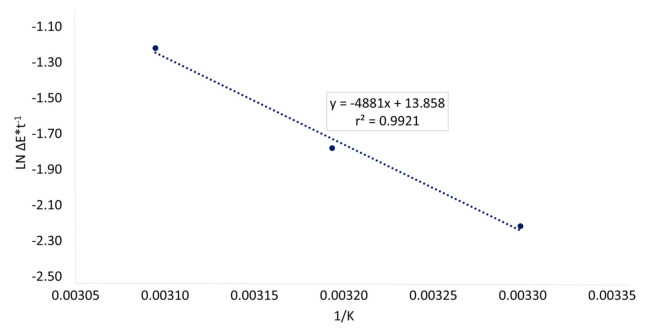
Arrhenius plot of pasteurized beetroot and apple mixed juice (PMIX), regarding total colour change (∆E) and storage time (t) as a function of storage temperature (1/K). The equation of the linear regression and determination coefficient (r^2^) are reported.

The natural logarithm of the rates of colour change of PMIX stored at 30, 40, and 50 °C were calculated and plotted against the temperature^−1^ [1/K]. Results are shown in Figure 5. The regression line was obtained, and the slope was used to predict the rate of colour change at different storage temperatures. 

**Figure 6:**
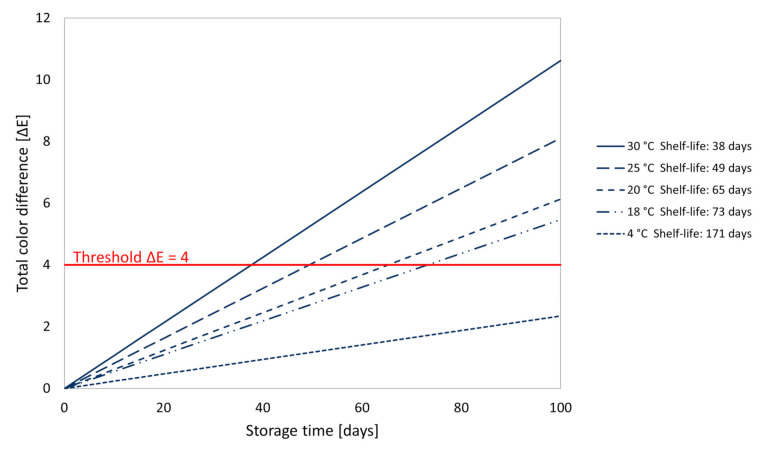
Predicted total colour difference (∆E) rated of pasteurized beetroot and apple mixed juice (PMIX) during storage at different temperatures (4, 18, 20, 25, and 30 °C) compared to the initial colour after processing. Predicted shelf-life corresponding to diverse storage temperatures is reported.

Through the rate of colour change at different storage temperatures, predictions of ∆E at different storage times were calculated. The predicted ∆E for PMIX stored at different temperatures (4, 18, 20, 25, and 30 °C) over time are shown in Figure 6.

These findings lead to a preliminary shelf-life, calculated based on the Arrhenius Model, of PMIX of 65 days, when stored at room temperature (20 °C) and of 171 days, when stored under refrigerated conditions (4 °C). Predicted shelf-life days corresponding to diverse storage temperatures are summarized in Figure 6.

**Figure 7:**
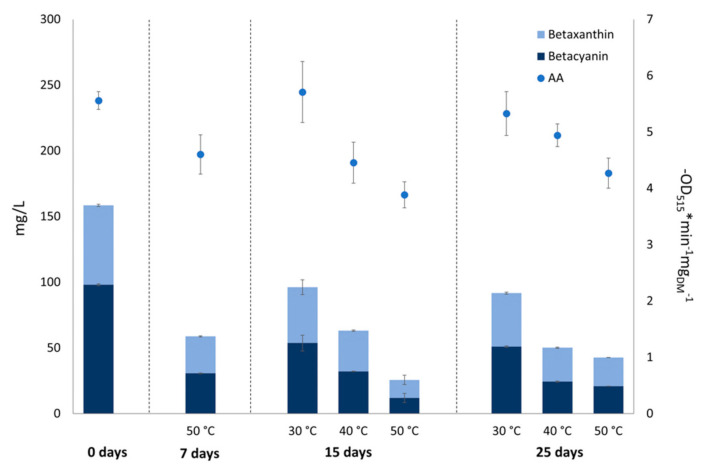
Content of betacyanins, betaxanthins, and antioxidant activity of pasteurized beetroot and apple mixed juice (PMIX) during storage at different temperatures, over a 25-day period.

Since colour in beetroot comes mainly from betalains which are, in turn, related to the antioxidant activity, among other functional properties, the effect of storage and temperature on betalains and on the antioxidant activity was evaluated. Concentration of betacyanins, betaxanthins, and antioxidant activity were determined in PMIX after storage at 30, 40, and 50 °C. Results are shown in Figure 7.

At the end of the storage period, a significant (*p* < 0.05) decrease in the betalain content was observed, ranging from 33 to 64% for betaxanthins and 48 to 73% for betacyanins. As expected, storage at the lowest temperature (30 °C) led to the smallest reduction in betalains (−42% of total betalain content after 25 days of storage at 30 °C versus −68% at 40 °C and −73% at 50 °C). In the storage conditions of absence of light used in this study, it would be expected, according to literature [12], that temperature would be the most relevant factor affecting the stability of betalains. A rapid degradation of betalains was observed during the first period of storage (7–15 days), after which the rate was lower up to 25 days. Surprisingly, in the PMIX stored at 50 °C, the betalain concentration was significantly (*p* < 0.05) lower at 15 days with respect to 25 days. Previous studies reported that, once oxidized, betalains release betalamic acid, which can condense with cyclo-L-3,4-dihydroxyphenylalanine (cyclo-DOPA) or amino acids to form de novo betalains [17]. This may explain the increased amount of betalains after 25 days of storage at 50 °C compared to 15 days, expecting that the above-mentioned chemical reactions could occur more rapidly at higher temperatures. Further experiments are required to assess the possibility that de novo formation of betalains at higher temperature could have occurred.

The content of betacyanins and betaxanthins decreased over time with a temperature-depending rate, with higher reduction for betacyanins. Similarly, another study showed a higher reduction of betacyanins than betaxanthins during storage of a beetroot and orange juice [32], and a recent work reported that betaxanthins and betacyanins were differently sensitive towards storage temperature, with betaxanthins being more stable than betacyanins [36]. On the contrary, previous works reported that betaxanthins are less stable than betacyanins upon heating [49,55].

While the antioxidant activity of PMIX was roughly constant at 30 °C, a significant reduction for the juice stored at 40 and 50 °C was observed (reduction of antioxidant activity of 11% and 23% after 25 days for PMIX stored at 40 and 50 °C, respectively). However, a trend toward increased antioxidant activity was observed in juices stored at 40 and 50 °C between 15 and 25 days of storage (Figure 7), which may be a result of the increased concentration of betalains observed in the same conditions.

Relationships among colour and betalain content were evaluated. A correlation coefficient of r = −0.77 could be observed between the total betalain content and ∆E; a correlation coefficient of r = −0.75 was found between the antioxidant activity and ∆E; a moderate positive correlation (correlation coefficient r = 0.65) between the betalain content and antioxidant activity was observed, as previously reported for beetroot and passion fruit juice [56]. These findings support the hypothesis that betalains may play a major role in the antiradical potential of this product, but they also point out that, after mixture with apple juice, a heterogenic product is generated in which many other compounds may interfere with the antioxidant potential.

**Figure 8:**
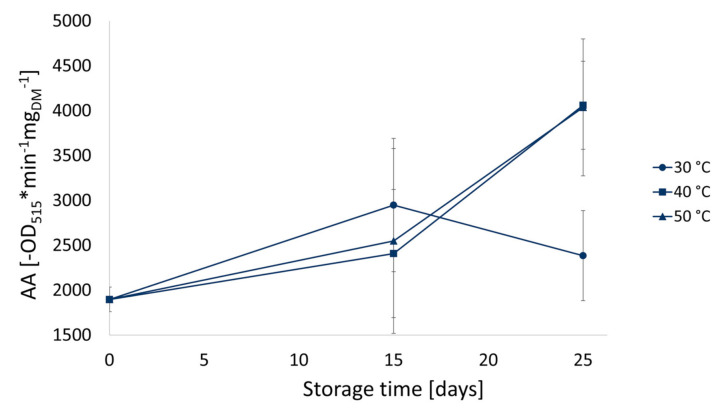
Antioxidant activity (AA) extrapolated based on the contribution of betalain compound present in pasteurized beetroot and apple mixed juice (PMIX) during storage at different temperatures (30, 40, and 50 °C).

Therefore, the AA assigned specifically to components coming from beetroot juice was extrapolated. The AA of PMIX was subtracted by that of PAJ and the difference—attributed to beetroot juice—was divided by the weight of total betalains. Results showing the contribution of betalain compounds to the AA in PMIX and their evolution during storage at different temperatures are shown in Figure 8.

Even though the AA and the betalain content in PMIX stored at 40 and 50 °C have overall decreased over time (Figure 7), the antioxidant potential related to the betalain content significantly (*p* < 0.05) increased during storage (Figure 8), confirming and emphasizing the above-mentioned tendency of AA to increase between 15 and 25 days of storage at 40 and 50 °C.

As already discussed, this increase may be ascribed to de novo formation of betalains [17], which may occur at higher storage temperatures, assuming a higher chemical reaction rate in this condition. Numerous reactions were reported concerning diverse food matrices that lead to an increase of AA as a result of processing or storage, including postharvest phenolic synthesis in fruits [57] and polymerization of polyphenols in processed food matrices [42], with consequent formation of partially polymerized phenols, that exhibit higher AA than non-oxidized ones. Beyond a certain level of molecular complexity (four monomer residues), the AA tends to decrease again [58,59].

Taking into account the numerous phenomena that can occur in a complex food matrix such as a beetroot and apple juice, including de novo betalain formation, polymerization of phenols, and interactions between molecules present in the two juices, an increase in antioxidant activity during storage under certain conditions can be plausible. Further studies with storage periods of at least 60 days are necessary to investigate these experimented findings.

## 4. Conclusions

Due to its attractive colour and content of bioactive compounds, beetroot represents an outstanding ingredient for innovative healthy functional beverages. In the present study, the effect of processing and storage on the quality of a natural product, from short-chain productions, consisting of beetroot and apple mixed juice was assessed.

The dilution of BJ with AJ successfully allowed to lower the pH below 4.5. The radical scavenging potential of BJ was found to be 10 times higher than that of AJ. Interestingly, the beetroot extraction step, though leading to a rise in temperature, did not substantially affect colour and antioxidant activity of the MIX. As expected, betalain content and antioxidant activity of BJ were reduced proportionally to the dilution with AJ. Thermal treatment of the mixed juice reduced betalains and determined colour changes, not influencing, however, its antioxidant potential.

Storage significantly affected the colour, the antioxidant activity, and the betalain content of PMIX. Nonetheless, after extrapolating the AA specifically attributed to betalains coming from beetroot juice, it was observed that the antioxidant activity of the residual betalains present in the PMIX stored at higher temperature increased over time, indicating that de novo formation of betalains may have occurred.

A provisional shelf-life of 65 days for the storage at room temperature (20 °C) was estimated. In order to verify whether the changes due to processing and storage are acceptable by consumers, the product should be further subjected to a sensory study that could implement the results based on instrumental technological step points.

## Figures and Tables

**Table 1:** Summary of the experimental dataset. Sample code, name, and a brief description are reported. For all pasteurized juices, the thermal treatment was carried out at 85 °C for 3 min in an oven with steam function.

Sample Code	Sample Name	Description
AJ	Apple juice	Cloudy apple juice purchased on the market
PAJ	Pasteurized apple juice	Apple juice (AJ) subjected to pasteurization
FBJ	Fresh beetroot juice	Raw beetroot juice obtained through a household juice extractor
BJ	Beetroot juice	Beetroot juice obtained by means of pilot-scale turboextractor
PBJ	Pasteurized beetroot juice	Beetroot juice (BJ) subjected to pasteurization
MIX	Beetroot and apple juice blend	Blended juice obtained by diluting beetroot juice (BJ; 15%) with apple juice (AJ; 85%)
PMIX	Pasteurized beetroot and apple juice blend	Beetroot and apple juice blend subjected to pasteurization

**Table 2:** List of the pairs of sample codes and related processing steps under investigation.

Comparison	Processing Step under Investigation
BJ-FBJ	Effect of processing-extraction
FBJ-PBJ	Effect of pasteurization on the quality of beetroot juice
AJ-PAJ	Effect of pasteurization of apple juice
MIX-PMIX	Effect of pasteurization of the beetroot and apple juice blend
AJ/BJ-MIX	Effect of addition of apple juice to beetroot juice

**Table 3:** Values (mean ± SD; n = 3) of pH and sugar content of apple juice (AJ), pasteurized apple juice (PAJ), fresh beetroot juice (FBJ), beetroot juice (BJ), pasteurized beetroot juice (PBJ), beetroot and apple mixed juice (MIX), and pasteurized beetroot and apple mixed juice (PMIX).

Sample Code	pH	Sugar Content (°Brix)
AJ	3.76 ± 0.01	12.77 ± 0.05
PAJ	3.92 ± 0.06	13.15 ± 1.13
FBJ	6.45 ± 0.03	15.72 ± 0.04
BJ	6.31 ± 0.01	10.37 ± 0.05
PBJ	6.10 ± 0.01	9.87 ± 0.05
MIX	4.16 ± 0.06	12.27 ± 0.08
PMIX	4.22 ± 0.06	12.15 ± 0.18

**Table 4:** CIE colour (L*, a*, and b*) values (mean ± SD; n = 3) of fresh beetroot juice (FBJ), beetroot juice (BJ), pasteurized beetroot juice (PBJ), beetroot and apple mixed juice (MIX), and pasteurized beetroot and apple mixed juice (PMIX).

Sample Code	CIE Colour Values		
	L*	a*	b*
FBJ	16.38 ± 0.17	4.78 ± 0.48	0.60 ± 0.11
BJ	16.12 ± 0.13	3.76 ± 0.58	0.63 ± 0.13
PBJ	16.75 ± 1.08	4.30 ± 0.35	1.09 ± 0.16
MIX	16.27 ± 0.07	3.81 ± 0.09	0.40 ± 0.10
PMIX	17.23 ± 0.22	8.96 ± 1.29	1.63 ± 0.36

**Table 5:** Colour difference (∆E) values (mean ± SD; n = 3) between fresh beetroot juice (FBJ) and beetroot juice (BJ), between BJ and beetroot and apple mixed juice (MIX), and between BJ and MIX and the corresponding pasteurized juices (PBJ and PMIX).

Sample Code	∆E
FBJ-BJ	1.07 ± 0.30
BJ-MIX	0.53 ± 0.35
MIX-PMIX	5.38 ± 1.29
